# Catastrophic health care expenditure among older people with non-communicable diseases in 11 European Union Member States

**DOI:** 10.1371/journal.pone.0346341

**Published:** 2026-04-29

**Authors:** Seda Kutluer, Milena Pavlova, Wim Groot

**Affiliations:** 1 Department of Health Services Research, CAPHRI, Maastricht University Medical Center, Faculty of Health, Medicine and Life Sciences, Maastricht University, Maastricht, the Netherlands; 2 Social Security Expert, Department of the General Directorate of Universal Health Insurance, Social Security Institution, Ankara, Türkiye; University of Bologna, ITALY

## Abstract

**Background:**

Out-of-pocket payments by patients with chronic diseases can result in catastrophic expenditures. This study examines how chronic disease burden, measured by the presence of any chronic condition such as diabetes, cancer, chronic lung disease, heart attack, stroke and high blood pressure and the number of chronic conditions are associated with the likelihood of catastrophic out-of-pocket payments among older adults in 11 European Union (EU) countries.

**Methods:**

The 2017 wave of the SHARE (Survey of Health, Ageing and Retirement in Europe) provides the most recent dataset suitable for our analysis. The sample size was 13,437. Probit regression models were estimated at 10%, 25% and 40% catastrophic health expenditure thresholds, controlling for demographic, socioeconomic and lifestyle characteristics, with standard errors clustered at the country level.

**Results:**

Our findings show that the number of chronic conditions was statistically significant association with catastrophic health expenditures across all thresholds, whereas the presence of any chronic condition was significant only at the 10% threshold and not at higher thresholds. Compared to older people in Spain, which country has the lowest level of catastrophic out-of-pocket health expenditures in the EU, older people in the Czech Republic, Greece, Italy and Poland more frequently experienced catastrophic health expenditure, whereas older people in Austria, Germany, Sweden, France, and Denmark exhibited lower risks.

**Discussion:**

Our findings demonstrate that multimorbidity, rather than the presence of a single chronic condition, drives catastrophic health expenditures among older adults. Older adults in the Czech Republic, Greece, Italy and Poland are more likely to experience catastrophic health expenditures compared to those in Spain, where out-of-pocket health expenditures are the lowest in the EU.

## 1. Introduction

Non-communicable diseases (NCDs) tend to be of long duration and are frequently the result of a combination of genetic, physiological, environmental and behavioral factors [1]. The increasing financial burden of NCDs has placed significant pressure on healthcare systems, affecting patients’ out-of-pocket expenditures and access to healthcare. NCDs may be grouped into cardiovascular diseases, cancers, chronic respiratory diseases and diabetes, according to the World Health Organization (WHO), and are also referred to as chronic diseases. Also, mental health problems are increasingly considered part of the broader NCDs agenda due to their close link with physical NCDs [1]. Globally, 74% of all deaths are caused by NCDs. In Europe, 91% of deaths are caused by NCDs [1]. Additionally, 80% of the disease burden in the EU is caused by NCDs [1,2].

The aging population in Europe significantly increases the use of healthcare services and medication expenses. For example, in 2018, individuals aged 65 and over accounted for 19.7% of the total population in the EU, and this proportion is expected to rise to 28.5% by 2050. Moreover, the increase in the older population leads to the rise in chronic diseases and long-term care needs, and increases the financial burden on the healthcare system.

Increased use of care and accumulated out-of-pocket payments cause a burden for individual patients as well, leading to catastrophic expenses. Catastrophic health expenditure refers to out-of-pocket healthcare payments that exceed a certain percentage of a household's income or consumption, leading to financial hardship. The WHO defines catastrophic health expenditure as occurring when a household's out-of-pocket payments surpass 10% of its total consumption or income. In this study, out-of-pocket expenditure means the total amount paid directly by individuals for health services during the past 12 months, without reimbursement from any public or private insurance. This includes costs for inpatient and outpatient care, prescribed medicines, nursing home or home-based care, aids, appliances and dental services. Together, these show the total financial burden individuals face when getting health care. In addition to the WHO’s standard 10% definition, some studies apply alternative “capacity-to-pay” measures, where catastrophic spending is defined using 25% or 40% thresholds after deducting basic living costs. These represent different methodological approaches rather than alternative WHO definitions. When households lack sufficient prepayment mechanisms or insurance coverage, they are more susceptible to experiencing catastrophic expenses. This draws attention to the importance of robust financial protection measures to prevent such economic burdens [3–6].

Out-of-pocket payments for healthcare vary greatly across EU countries. While some, like Sweden and Germany, offer broad coverage and limit patient payments, others, such as Bulgaria and Greece, report widespread informal or additional payments. These costs often create a financial burden, especially for older adults and those with chronic diseases. In some countries, complementary insurance helps reduce the burden, but access depends on individuals’ ability to pay [7–18].

Recent international evidence underscores the strong association between chronic disease burden and catastrophic health expenditure. For example, a recent cohort study examining older Chinese households showed that both single and multiple chronic conditions substantially increased the risk of catastrophic health expenditures, even among individuals enrolled in basic medical insurance schemes [19]. Beyond individual-level determinants, comparative evidence from the “Health Systems in Transition” country reports highlight substantial variation across European health systems in co-payment rules, out-of-pocket payment levels, and the extent of financial protection for vulnerable groups [20,21]. These system-level differences help explain the cross-national variation in catastrophic health expenditures between European countries. Previous studies have also shown that older adults and individuals with chronic diseases face higher financial risks [22]. By combining insights from different studies, our research provides new cross-country evidence from 11 EU member states and contributes to global discussions on how NCDs and health system factors together affect households’ risk of catastrophic health expenditures.

Across Europe, substantial differences exist in the structure and financing of national health systems, which directly influence the level of out-of-pocket payments. Countries such as Sweden, Denmark, and Italy operate mainly universal, tax-financed National Health Service (NHS) systems with broad population coverage and relatively low cost-sharing. In contrast, Germany, France, Belgium, and Austria rely on social health insurance models funded through mandatory contributions but with varying co-payment requirements.

Other countries included in our analysis, such as Greece, Poland, Spain, and the Czech Republic, have mixed or insurance-based systems combining public financing with higher private spending, offering less financial protection for vulnerable groups. These structural differences, including co-payment policies, exemptions, and access to supplementary insurance, help explain why out-of-pocket and catastrophic health expenditures vary between countries [23,24].

Given the diversity of out-of-pocket arrangements in the EU, their burden on older adults with chronic diseases needs to be clarified. This population group is suffering more from chronic diseases than younger population groups. Accumulated out-of-pocket payments by patients with chronic diseases can result in catastrophic expenditures for the household.

Previous research has highlighted the significant financial burden of out-of-pocket health expenditures on individuals with chronic diseases across Europe and beyond. These studies show that out-of-pocket costs, particularly for medicines and outpatient services, often lead to catastrophic health spending, especially among low-income and older adults populations [25–29]. Regional variations have also been documented, with countries like Greece, Portugal, and Poland reporting higher out-of-pocket spending, while others, such as the Netherlands and Luxembourg, have lower levels [26,28]. In low- and middle-income countries, the financial impact is even more severe, disproportionately affecting vulnerable groups [27].

Despite this growing body of literature, most studies focus on quantifying the burden rather than exploring the underlying factors driving these variations across countries. Furthermore, little attention has been given to the specific role of system-level factors, such as health financing structures or cost-sharing mechanisms, in shaping these disparities [26,30–32]. Addressing this gap, this study investigates the determinants of out-of-pocket health expenditures among patients with chronic conditions, offering new insights into potential policy instruments to reduce financial hardship by following the methodology of the previous study [27].

According to the WHO, the five main categories of NCDs include cardiovascular diseases, cancers, chronic respiratory diseases, diabetes, and mental and neurological disorders. In our study, we focused on six specific NCDs, such as diabetes, cancer, heart attack, stroke, chronic lung disease, and high blood pressure, which represent the first four WHO-defined categories. Mental health conditions were not included due to the lack of reliable data in the SHARE dataset. Our study builds upon this existing evidence by updating and expanding the scope to 11 EU member states. By examining cross-country differences, the study aims to contribute to policy discussions on how to address this issue [26,30]. This study highlights the heavy burden of healthcare expenditures on low-income and vulnerable groups. Based on these findings, policies can be developed to strengthen financial protection measures by adjusting co-payment structures or expanding support mechanisms for at-risk populations. Across Europe, healthcare financing reforms are needed to reduce the economic burden of chronic diseases [27,31,32].

The most recent data suitable for our analysis is the 2017 SHARE dataset, which contains data about out-of-pocket health expenditure among older adults in 11 European member states (Austria, Germany, Sweden, Spain, Italy, France, Denmark, Greece, Belgium, Czech Republic and Poland). We included out-of-pocket payments for dental care, medical aids, appliances and physical therapy in addition to inpatient, outpatient, medication and nursing home payments. Considering total out-of-pocket spending, instead of spending for specific chronic diseases, allows us to better capture the overall financial burden on households since healthcare expenditures for different illnesses are often interconnected. Catastrophic health expenditures can be measured using different approaches. Some methods compare out-of-pocket payments to total health expenditures or household income, while others adjust for essential living costs, such as food and housing expenses, when assessing financial burden.

In this study, we expect higher catastrophic expenditure rates in countries with lower (government) health expenditures, higher private (out-of-pocket) payments, and lower exemptions for older adults and chronic patients. S1 Table presents the differences in health expenditure patterns among the 11 EU member states included in the study. Based on the data for 2017 (the year of data collection), we expect higher catastrophic expenditures in the Czech Republic and Poland (lowest total health expenditure as a percentage of GDP (Gross Domestic Product)), Italy (lowest government expenditure on health as a percentage of GDP) and Greece (highest private and out-of-pocket expenditures as a percentage of total health expenditure). As mentioned earlier, Poland and Greece are also countries with limited exemptions for older adults and people with chronic diseases.

The financial burden of out-of-pocket payments among older adults with chronic conditions (diagnosed with diabetes, cancer, chronic lung disease, heart attack, stroke, and high blood pressure) varies substantially across health systems. However, comprehensive comparative evidence on how different NCDs contribute to catastrophic health expenditure across EU member states remains limited. This study aims to assess how having any chronic condition and the number of chronic conditions influence the likelihood of experiencing catastrophic health expenditure among older adults in 11 EU countries. It seeks to answer the following research question: How do chronic conditions and multimorbidity affect the likelihood of catastrophic health expenditure among older adults across countries?

## 2. Methods

The analysis is based on Wave 7 of the SHARE survey, conducted in 2017, which is the most recent wave that includes detailed data on out-of-pocket health expenditures. Although Waves 8 and 9 have since been released, they do not contain comparable out-of-pocket expenditure data, which is critical for estimating catastrophic health spending. Wave 7 was conducted in 28 countries, including all EU member states in 2017. The anonymized SHARE Wave 7 data were accessed for research purposes on 1 January 2025 via the SHARE ERIC (European Research Infrastructure Consortium) data portal. This survey was designed by the Health, Ageing and Retirement in Europe (SHARE) research team and data collection was carried out by national survey agencies and research institutions in each participating country. Sampling was based on a probabilistic, multi-stage design to ensure the representativeness of the population across Europe [33–36]. The data collection method was based on face-to-face interviews and focused on life histories in the areas of health, family, housing and employment. The data collection in the Netherlands was completely different from that in other countries, and therefore, these data were not included in the main dataset for 2017. Also, data on out-of-pocket payments were not collected in all countries [35–37]. Therefore, our final analytical sample included 11 EU member states (Austria, Germany, Sweden, Spain, Italy, France, Denmark, Greece, Belgium, the Czech Republic, and Poland). This study was based on secondary analysis of anonymized SHARE Wave 7 data. No additional ethical approval was required, and informed consent was not applicable.

To select data for our analysis, we merged different modules of the 2017 SHARE dataset, including modules on health, healthcare, physical activity, household income, behavioral risks, demographics and networks. When applicable, we also used the imputations provided in the dataset and explained in the SHARE guidelines [38]. In SHARE Wave 7, there is equivalized household income measure (the variable thinc2). Therefore, the analysis is based on income that has been adjusted using an equivalence scale. The survey dataset allowed us to include data on respondents’ expenditure for inpatient care, outpatient care, medication, nursing home, dental, medical aids, appliances and physical therapy, as well as data on whether the respondents had been diagnosed with NCDs such as diabetes, cancer, heart attack, stroke, chronic lung disease and high blood pressure. The dependent variable is catastrophic health expenditures for three different threshold levels [39,40], namely 10%, 25% and 40%. As one of the independent variables, we generated a new variable (binary variable), called ‘any condition’, which presents whether an individual has reported having at least one chronic disease. We used it as one of the explanatory variables in the first set of models. In the second set of models, this explanatory variable was replaced with a variable with indicated the number of chronic diseases an individual has reported (called ‘number of chronic conditions’). Also, we created an indicator variable equal to 1 if the respondent was recorded as deceased in Wave 8 (or no longer present in the panel due to death) and 0 otherwise. This newly generated variable was included in each model as one of the explanatory variables in order to capture potential end-of-life health expenditures that may increase out-of-pocket health expenditures in Wave 7 [41]. All other explanatory variables are from Wave 7 except for the ‘deceased individual’ variable defined above. In particular, we included health-risk variables and socio-demographic variables that appeared relevant in previous research [42]. Other independent variables are gender, age, education level, household size, number of children, expenditure percentile groups, smoking, alcohol consumption, body mass index, physical activity and cholesterol status. These variables were included to capture key demographic, socioeconomic and lifestyle factors that may influence the likelihood of experiencing catastrophic health expenditure.

### 2.1. Outcome variable: Catastrophic expenditure

To determine the prevalence of catastrophic health spending, we need to calculate the proportion of households whose out-of-pocket health expenditures exceed a specified threshold of their total household expenditure or income [39,40]. We executed two sets of probit models (as explained above) with clustered standard errors and country fixed effects (using country dummies with one base category). The dependent variable is catastrophic expenditures for all chronic diseases with 10% thresholds. Furthermore, we added two other models with 25% and 40% thresholds for all catastrophic expenditures. In sum, six probit models were estimated. Spain was used as the reference country category.

There are different methods for calculating the health expenditure-wealth ratio. The budget share method, used in the Sustainable Development Goals (SDG), defines catastrophic spending as out-of-pocket payments exceeding 10% or 25% of total household income or expenditure. Other methods focus on the capacity to pay, meaning they subtract essential expenses before calculating health spending. “Actual food spending method” defines catastrophic spending as out-of-pocket payments exceeding 25% or 40% after deducting actual food costs. “Partial normative food spending method” uses a 40% threshold after subtracting a standard amount for necessary food expenses. “Normative spending on food, housing, and utilities method” considers out-of-pocket catastrophic if it exceeds 40% after subtracting food, rent, and utility costs [39]. If many households exceed this threshold, it indicates that healthcare services are not financially protective. A high prevalence rate suggests that health expenditures can push people into poverty and that there are significant gaps in financial protection within the health system.

**Fig 1:**
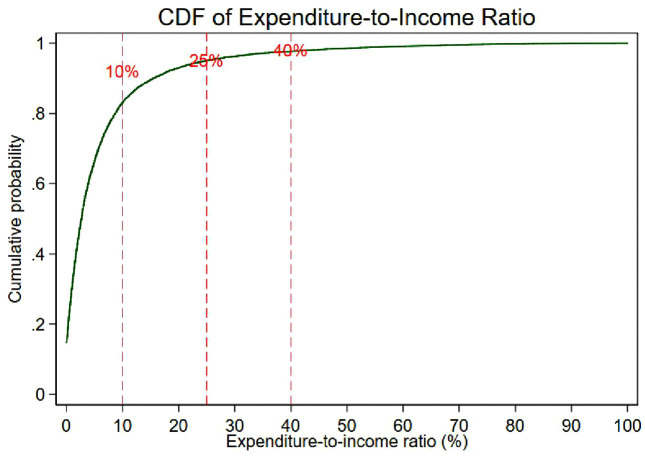
Cumulative frequency of catastrophic health expenditures across different thresholds (0–40%) in 11 EU countries.

We used the budget share method by dividing the respondents’ total out-of-pocket payments for health care (health expenditure) by the total respondents’ household income per capita (wealth). Income was used only as a denominator in the outcome variable and was not included as a separate explanatory variable in the models. We used income as a wealth indicator because we had no complete data for household expenditure. This income variable is equivalized household income measure. Fig 1 shows the cumulative frequency of catastrophic expenditure that we calculated for different thresholds, from 5% to 40%. As can be seen in the figure, frequencies are notably high in the “up to 5%” and “more than 40%” spending groups, and they remain relatively uniform in the mid-ranges (10–35%). This means that households are divided into different expenditure groups regarding healthcare spending. While some households spend very little, others face significant financial difficulties due to extremely high healthcare costs. Although there is a balance at moderate levels, financial protection mechanisms are insufficient to protect households facing the highest out-of-pocket payments. For our analysis, we used the 10% threshold, which is recommended for the budget share method [40,43] and was used in previous studies [26,27,30,31,42].

To provide a clearer depiction of the distribution of catastrophic health expenditures, Fig 1 presents the cumulative distribution function of the expenditure-to-income ratio, with the 10%, 25% and 40% thresholds highlighted. Although cumulative frequency graphs can be more complex to interpret when comparing multiple countries and thresholds simultaneously, we chose this format to provide an overall visual distribution of financial burden due to out-of-pocket payments.

### 2.2. Explanatory variables: Socio-demographic, lifestyle factors and country variables

Explanatory variables in our analysis were socio-demographic factors such as gender, age, years of education, number of children, household expenditure percentiles (including five groups), household size and lifestyle risk factors such as smoking, alcohol consumption, body mass index, physical activity and cholesterol status [44–60]. Total household health expenditure was divided into five equal-sized groups (quintiles) to allow for comparisons across expenditure levels. Spain was used as the baseline country for comparison since the catastrophic health spending level was the lowest, according to WHO data [46]. We used country dummy variables for the other 10 countries.

Explanatory variables included a combination of binary, categorical, and continuous variables. Binary variables were ever smoked, cholesterol status, and gender (coded as 1 = male, 0 = female, with female as the reference category). Categorical variables included body mass index (BMI) category, household health expenditure groups, and country dummies. Continuous variables were age, years of education, household size, and number of children. Each variable was selected based on prior literature and theoretical relevance to cardiovascular risk and health expenditure.

### 2.3. Probit regression models

We estimated six probit regression models to assess the relationship between chronic conditions and the likelihood of experiencing catastrophic health expenditure among older adults. As detailed in the previous section, the first set of models used a binary indicator for having at least one chronic disease, while the second set of models included the number of chronic conditions as the main explanatory variable. In each set, three regression models were estimated, using three different threshold levels of catastrophic health expenditures (10%, 25%, and 40%) as the dependent variable. All models incorporated demographic, socioeconomic, and lifestyle covariates, along with country fixed effects and clustered standard errors at the country level.

After estimating the probit models, several post-estimation diagnostics were conducted to assess model specification, fit, and robustness for each of six probit models. First, the link test showed that the model form was appropriate. Second, the Receiver Operating Characteristic Curve (ROC) showed that all models could clearly distinguish between people with and without catastrophic health expenditure. Third, the Hosmer–Lemeshow test indicated that the model fits the data well. Fourth, the Variance Inflation Factor (VIF) values were low, meaning there was no multicollinearity problem. Finally, the marginal effects helped interpret how each variable affects the probability of experiencing catastrophic expenditure. Overall, the diagnostics confirm that the models are well specified, show good fit, do not suffer from multicollinearity issues, and produce meaningful marginal effects.

## 3. Results

Descriptive statistics for outcome variables and explanatory variables are presented in S2 Table. Out-of-pocket payments for different types of services and NCDs are summarized in S3 Table.

The total sample size is 13,437. Overall, 45% of the sample is male and 55% is female. Although the SHARE survey includes individuals aged 50 and older, the out-of-pocket health expenditure data used in this study were only collected for respondents aged 66 and above in Wave 7. As a result, our analytical sample starts at age 66. The average age is 76 years. About 13% of respondents experienced catastrophic health spending. High blood pressure is the most prevalent condition, affecting 52% of respondents. Diabetes is reported by 17% of the sample. Heart attack and chronic lung disease have a prevalence of 16% and 7%, respectively. Cancer and stroke are the least common conditions, affecting 5% of respondents. The sample includes respondents from 11 European countries. 43% of respondents reported ever smoking. Alcohol consumption ranges from 0 (not at all) to 5 (excessive consumption), with 65% reporting high consumption. Physical activity is reported by 81% of respondents. High cholesterol is present in 25% of the sample. The average BMI is 27, with values ranging from 13 to 56. Catastrophic health care expenditure is measured using three alternative thresholds. At the 10% threshold, 13% of the sample experience catastrophic health expenditure, while 87% do not. When the threshold is increased to 25%, the share of individuals experiencing catastrophic expenditure declines to 4%, and further decreases to 2% at the 40% threshold, indicating that severe levels of catastrophic spending affect a smaller proportion of the population. Chronic health conditions are measured in two ways. The number of chronic conditions ranges from zero to five, capturing the extent of multimorbidity. In addition, a binary indicator shows that 52% of respondents report having at least one chronic condition, while 48% report none. Mortality during the observation period is relatively low, with 5% of respondents reported as deceased by wave 8 (Table 1).

**Table 1:** Results of Probit Models with different threshold levels: % 10, % 25 and % 40 for Model 1 and 2.

	Catastrophic effects of health care costs (Model 1)						Catastrophic effects of health care costs (Model 2)					
Dependent Variable	Catastrophic effects of health care expenditure for all sample with %10 threshold (1 = yes; 0 = no)		Catastrophic effects of health care expenditure for all sample with %25 threshold (1 = yes; 0 = no)		Catastrophic effects of health care expenditure for all sample with %40 threshold (1 = yes; 0 = no)		Catastrophic effects of health care expenditure for all sample with %10 threshold (1 = yes; 0 = no)		Catastrophic effects of health care expenditure for all sample with %25 threshold (1 = yes; 0 = no)		Catastrophic effects of health care expenditure for all sample with %40 threshold (1 = yes; 0 = no)	
Explanatory Variables	B	SE	B	SE	B	SE	B	SE	B	SE	B	SE
Having at least one chronic condition (Model 1)	0.167**	0.065	0.128	0.08	0.076	0.076	—	—	—	—	—	—
Number of chronic conditions (Model 2)	—	—	—	—	—	—	0.160***	0.045	0.097***	0.034	0.071**	0.03
Deceased by wave 8	0.071	0.047	0.191**	0.092	0.162*	0.085	0.056	0.049	0.182**	0.091	0.156*	0.083
Ever smoked	0.033	0.033	0.01	0.056	0.016	0.057	0.023	0.034	0.003	0.054	0.009	0.055
Alcohol consumption	0.035	0.023	0.016	0.039	0.005	0.045	0.031	0.022	0.013	0.039	0.003	0.046
Body mass index	0.01	0.02	0.003	0.029	−0.012	0.032	−0.004	0.018	−0.004	0.029	−0.018	0.032
Physical activity	−0.184***	0.043	−0.259***	0.035	−0.335***	0.057	−0.147***	0.048	−0.235***	0.039	−0.315***	0.061
High level of cholesterol	0.088**	0.04	0.081	0.065	0.091	0.06	0.046	0.033	0.057	0.062	0.07	0.064
Gender	−0.200***	0.035	−0.269***	0.042	−0.323***	0.047	−0.216***	0.035	−0.273***	0.044	−0.328***	0.048
Age	0.024***	0.002	0.018***	0.005	0.018***	0.006	0.023***	0.002	0.018***	0.005	0.017***	0.006
Years of education	−0.007	0.008	−0.012***	0.004	−0.012*	0.007	−0.006	0.008	−0.012	0.007	−0.011	0.007
Number of children (age < 18 years)	0.011	0.014	0.002	0.032	−0.029	0.032	0.009	0.014	0.002	0.032	−0.029	0.031
Household Expenditure percentiles	0.345***	0.032	0.459***	0.054	0.515***	0.077	0.345***	0.033	0.457***	0.055	0.513***	0.078
Austria	−0.195***	0.058	−0.344***	0.077	−0.615***	0.082	−0.201***	0.058	−0.350***	0.077	−0.619***	0.084
Belgium	−0.118	0.087	−0.499***	0.092	−0.904***	0.115	−0.103	0.087	−0.492***	0.093	−0.898***	0.112
Czech Republic	0.345***	0.042	0.331***	0.076	0.305***	0.11	0.329***	0.044	0.319***	0.077	0.292***	0.109
Denmark	−0.425***	0.102	−0.835***	0.108	−1.347***	0.133	−0.432***	0.101	−0.841***	0.109	−1.348***	0.133
France	−0.444***	0.085	−0.550***	0.09	−0.874***	0.106	−0.439***	0.085	−0.549***	0.09	−0.878***	0.106
Germany	−0.483***	0.088	−0.736***	0.095	−0.951***	0.094	−0.506***	0.087	−0.747***	0.095	−0.964***	0.096
Greece	0.437***	0.031	0.001	0.053	−0.145**	0.071	0.428***	0.031	−0.007	0.053	−0.154**	0.069
Italy	0.160***	0.023	−0.047	0.031	−0.185***	0.032	0.166***	0.024	−0.044	0.031	−0.183***	0.032
Poland	1.264***	0.062	1.020***	0.151	0.861***	0.24	1.243***	0.066	1.000***	0.153	0.845***	0.24
Sweden	−0.149*	0.082	−0.828***	0.102	−1.180***	0.121	−0.155*	0.081	−0.830***	0.103	−1.181***	0.122
Constant	−3.810***	0.205	−3.969***	0.358	−4.052***	0.457	−3.763***	0.204	−3.926***	0.357	−4.022***	0.453
R-square	0.136	—	0.155	—	0.246	—	0.142	—	0.198	—	0.247	—
Number of Observations	10,205	—	10,205	—	10,205	—	10,205	—	10,205	—	10,205	—
Post Estimation Tests	value		value		value		value		Value		value	
Linktest (_hat) p-değeri	<0.001		<0.001		<0.001		<0.001		<0.001		<0.001	
Linktest (_hatsq) p-değeri	0.104		0.385		0.584		0.31		0.497		0.569	
AUC (ROC)	0.755		0.832		0.879		0.7596		0.8334		0.8798	
GOF (Hosmer–Lemeshow)	χ²(8)=8.21		χ²(8)=7.53		χ²(8)=13.07		χ²(8)= 7.92		χ²(8)=6.15		χ²(8)=13.33	
GOF p-değeri	0.413		0.48		0.109		0.4414		0.6307		0.1009	
LR χ² test	1285.98		852.71		668.92		1347.81		861.76		672.69	
LR χ² p-değeri	<0.001		<0.001		<0.001		<0.001		<0.001		<0.001	
Number of observations	10,205		10,205		10,205		10,205		10,205		10,205	

Significance levels: * p < 0.1, ** p < 0.05, *** p < 0.01.

Table 1 presents the results of probit regression models examining the determinants of catastrophic health care expenditures among older individuals at three different thresholds (10%, 25%, and 40%). Two model specifications are reported: The first set of models captures the presence of having one chronic condition, while the second set of models reflects multimorbidity through the number of chronic conditions.

Being deceased by wave 8 is associated with higher catastrophic health expenditures, especially at higher thresholds. Smoking, alcohol consumption, and body mass index are not significantly related to catastrophic health expenditures. In contrast, physical activity is consistently associated with a lower probability of catastrophic health expenditures in all models. High cholesterol shows a positive relationship in some models, although this effect is not consistent across thresholds.

Women are more likely to experience catastrophic health expenditures than men, and older age is associated with a higher risk in all models. Years of education and the number of children do not show a strong or consistent relationship with catastrophic health expenditures. Household expenditure percentiles are positively and strongly associated with catastrophic health expenditures in all specifications.

There are clear differences across countries. Compared with the reference country, Austria, Germany, France, Denmark, and Sweden show lower probabilities of catastrophic health expenditures in most models. Poland shows a higher risk across all thresholds, while the Czech Republic also has a higher probability of catastrophic health expenditures. Italy and Greece show mixed results depending on the threshold, and Belgium shows lower risk mainly at higher thresholds. Post-estimation tests indicate that all models are statistically significant, have good ability to distinguish outcomes, and show acceptable model fit.

Overall, the explanatory power of the models increases with higher thresholds, indicating that severe catastrophic health expenditures are more closely linked to underlying health and structural factors.

Table 2 shows the association between different health expenditure indicators and both the probability of catastrophic health spending and the likelihood of having specific NCDs. The findings indicate that higher total health expenditure as a percentage of GDP and greater public health expenditure are generally associated with a lower risk of catastrophic spending across most NCDs. In contrast, higher out-of-pocket payments and a greater share of private health expenditure tend to increase the risk. The models also suggest that financial health system characteristics influence the probability of being diagnosed with NCDs, with varying effects across disease types. Notably, diabetes and high blood pressure show the strongest associations with catastrophic spending risk.

**Table 2:** Correlation between variables (Pearson Correlation).

	Total health expenditure as a percentage of GDP	Out-of-pocket payments as a percentage of total health expenditure	Public health expenditure as a percentage of total health expenditure	Private health expenditure as a percentage of total health expenditure	Out-of-pocket payments as a percentage of private health expenditure
P (Expected catastrophic expenditure| diabetes = 1), **R² = 0.2203**	−0.231***	0.161***	−0.175***	0.140***	0.033***
Probability of diabetes = 1	−0.087***	0.04***	−0.075***	0.035***	0.0102
P (Expected catastrophic expenditure| cancer = 1), **R² = 0.0797**	−0.0375***	0.03***	−0.028***	0.029***	0.002
Probability of cancer = 1	0.025***	−0.035***	0.029***	−0.026***	−0.021**
P (Expected catastrophic expenditure| chronic lung disease = 1), **R² = 0.2274**	−0.044***	0.044***	−0.043***	0.019**	0.045***
Probability of chronic lung disease = 1	0.002	−0.003	0.018	−0.012	0.005
P (Expected catastrophic expenditure| heart attack = 1), **R² = 0.2264**	−0.1302***	0.096***	−0.080***	0.095***	−0.006
Probability of heart attack = 1	−0.041***	0.022**	−0.02**	0.041	−0.043***
P (Expected catastrophic expenditure| stroke = 1), **R² = 0.2932**	−0.100***	0.071***	−0.060***	0.063***	0.001
Probability of stroke = 1	−0.0336***	0.016	−0.007	0.016	−0.002
P (Expected catastrophic expenditure| high blood pressure = 1), **R² = 0.1701**	−0.325***	0.258***	−0.274***	0.215***	0.067***
Probability of high blood pressure = 1	−0.139***	0.123***	−0.1***	0.103***	0.041***
P (Expected catastrophic expenditure non-communicable diseases all = 1, R² = 0.0989	−0.302***	0.276***	−0.317***	0.209***	0.115***
Probability of non-communicable diseases all = 1	−0.098***	0.112***	−0.0789	0.107***	0.008

Significance levels: * p < 0.1, ** p < 0.05, *** p < 0.01.

Number of observations = 11 (country-level averages).

Abbreviations: GDP = Gross Domestic Product, R² = R-square.

## 4. Discussion

This discussion is based on the empirical findings obtained from six probit regression models estimating the likelihood of catastrophic health expenditure among individuals aged 50 years and above in 11 European Union countries using SHARE Wave 7 data [35]. The results provide consistent evidence that chronic disease burden is a key determinant of catastrophic health expenditure, with multimorbidity emerging as the principal driver of financial vulnerability [25].

The findings show that both individual health conditions and differences between national health systems play an important role in explaining catastrophic health expenditures [61,62]. The strong effect of the number of chronic conditions suggests that overall disease burden, rather than a single diagnosis, is a major source of financial risk for households. The higher risk observed among individuals deceased by wave 8 reflects the high medical costs often faced near the end of life.

Health-related behaviors show mixed effects. While smoking, alcohol use, and body mass index are not directly linked to catastrophic health expenditures, physical activity appears to reduce the risk, possibly by improving overall health and reducing the need for expensive medical care.

Demographic factors are also important. Women and older individuals face a higher risk of catastrophic health expenditures, which may be related to greater health care use, longer life expectancy, and differences in economic resources.

The strong country differences highlight the importance of health system organization and financing. Countries such as Germany, France, Denmark, Sweden, and Austria appear to offer better financial protection, likely due to stronger health insurance coverage and lower out-of-pocket payments. In contrast, the higher risks observed in Poland and the Czech Republic suggest weaker financial protection. The mixed results for Italy and Greece indicate differences in how health systems perform across expenditure thresholds. Overall, the post-estimation results support the reliability of the findings and suggest that the main conclusions are robust across model specifications.

Our results are in line with previous studies and highlight the need for effective public health policies to reduce NCDs treatment costs and financial burden across EU member states [25–41]. Countries with universal healthcare, such as Germany, France, Sweden, and the Netherlands, have lower out-of-pocket health expenditures, reducing financial hardship. Countries with less comprehensive healthcare systems, such as Poland, Romania, Bulgaria, and Greece, have higher out-of-pocket payments, increasing the risk of catastrophic health spending, particularly for low-and middle-income households. Aging populations and multimorbidity further increase healthcare costs [25]. Furthermore, a comparison with one of the previous studies that used a similar methodology and dataset also shows a rising trend in these expenditures over time, emphasizing the ongoing shortcomings in financial protection mechanisms within healthcare systems [41].

The persistent disparities in catastrophic health expenditures across European regions highlight the impact of healthcare system structures, financing mechanisms, and cost-sharing policies on financial burden. In particular, Central and Eastern European countries, such as Poland, Greece, and the Czech Republic, consistently report higher catastrophic health expenditures, indicating potential shortcomings in public health financing and insurance coverage [61]. By contrast, Western European countries like Germany and Austria exhibit lower catastrophic health expenditures in certain disease categories, suggesting that well-developed financial protection policies can mitigate economic distress [62]. Lower-income households bear a disproportionate burden, further demonstrating the inequitable nature of out-of-pocket healthcare expenses and underscoring the urgent need for fairer cost-sharing policies [63]. Moreover, older individuals face a higher likelihood of catastrophic health expenditures, suggesting that current financial protection mechanisms may be insufficient to cover healthcare costs.

To mitigate catastrophic health expenditure, especially among individuals affected by NCDs, several regulatory and policy-level interventions are essential. First, universal health insurance must guarantee equitable access to essential healthcare services. In many countries, individuals with NCDs face disproportionate financial burdens due to the chronic nature of their illnesses and the long-term need for medications and monitoring. Expanding insurance coverage to include a broader range of health services, such as diagnostic tests, medications, and rehabilitation, is critical to minimizing out-of-pocket expenditures [64,65].

Moreover, cost-sharing mechanisms, such as co-payments, deductibles, and user fees, need to be limited, especially for low-income populations and those suffering from NCDs. Studies show that even modest co-payments can hinder care-seeking behavior an amplify health inequalities [66]. Targeted financial assistance programs, including health equity funds and conditional cash transfers, may support disadvantaged groups and protect vulnerable populations from current health expenditures. These programs have been shown to improve healthcare access and reduce financial hardship, particularly in low- and middle-income countries [67].

Expanding health assistance through direct subsidies or voucher schemes can further enhance access to vital services. These interventions not only reduce out-of-pocket spending but also contribute to a more equitable and efficient healthcare system. Improving the delivery of healthcare services is also essential to reduce the burden of current health expenditures. Strengthening primary healthcare systems through investments in family physicians, community health centers, and preventive care—can reduce unnecessary hospital visits and associated costs [68]. Increased public health expenditure targeting NCDs is another crucial strategy. Investments in early screening, preventive interventions, and public awareness campaigns can lead to early detection and improved disease management, thereby reducing the long-term economic burden on individuals and the health system [68].

This study has several limitations. Data availability was a key constraint, as the SHARE dataset does not provide data on out-of-pocket payments for all EU member states. Wave 7 dataset is the most recent year data for which out-of-pocket payments data are available. Thus, we were not able to explore the most recent situation. Additionally, some total household expenditure data had missing values at the individual level. As a result, we could not apply an alternative method for calculating catastrophic health expenditures, which prevented us from incorporating wealth-based expenditure calculations. We only took into account total household income while calculating catastrophic health expenditures. In addition, we excluded the “origin variable” due to missing data. Furthermore, this study does not account for individuals who require healthcare services but are unable to afford them due to low incomes. We recommend that future research consider foregone care costs in order to efficiently assess the extent to which individuals are affected by catastrophic health expenditures. Therefore, it is also essential to consider in comparative analyses that each country has a distinct health insurance system and different levels of coverage.

## 5. Conclusion

This study finds that multimorbidity significantly increases the risk of catastrophic health expenditures across European countries. It also shows that catastrophic health expenditures are mainly influenced by chronic disease burden, age, gender, and differences in health system protection across countries. Stronger health financing and social protection systems can reduce households’ exposure to catastrophic health care costs, especially for vulnerable groups.

These results underline the urgent need to strengthen financial protection mechanisms for individuals with NCDs. Future research should incorporate data on unmet healthcare needs and country-specific insurance features to provide a more comprehensive understanding of financial hardship. Comparative analysis using more recent and detailed data would further support the development of equitable and effective health policies. In conclusion, an integrated approach combining universal coverage, cost reduction, targeted financial support, primary care strengthening, and drug price regulation can significantly alleviate the burden of current health expenditures among individuals with NCDs and improve the financial protection capacity of health systems.

## Supporting information

S1 Table Macro Indicators on health expenditure in 11 EU member states. (PDF)

S2 Table Descriptive Statistics for Total Sample (N = 13.437). (PDF)

S3 Table Out-of-pocket payments for different types of services and different NCDs (all amounts are expressed in Euros). (PDF)
