# Supplementary material for: Catastrophic health care expenditure among older people with non-communicable diseases in 11 European Union Member States
Source: PLoS One. 2026 Apr 29;21(4):e0346341. doi: 10.1371/journal.pone.0346341 (PMC13127918; doi:10.1371/journal.pone.0346341)
Supplement: S1 Table — (PDF) [file pone.0346341.s001.pdf]

S1 Table. Macro indicators on health expenditure in 11 EU member states

	**Total health expenditure as percentage of GDP**		**Government expenditure on health as a percentage of GDP**		**Private health expenditure as a percentage of total health expenditure**		**Out-of-pocket as a percentage of total health expenditure**		**Out-of-pocket expenditure as a percentage of private expenditure**	
**Countries**	**2017**	**2021**	**2017**	**2021**	**2017**	**2021**	**2017**	**2021**	**2017**	**2021**
Austria	10%	12%	8%	9%	26%	22%	19%	16%	73%	73%
Belgium	11%	11%	8%	9%	23%	22%	18%	18%	79%	80%
The Czech Republic	7%	9%	6%	8%	16%	14%	15%	13%	91%	92%
Denmark	10%	11%	9%	9%	16%	15%	14%	13%	85%	85%
France	11%	12%	8%	9%	24%	24%	10%	9%	40%	37%
Germany	11%	13%	9%	10%	22%	21%	13%	12%	58%	58%
Greece	8%	9%	6%	5%	48%	41%	35%	33%	73%	82%
Italy	9%	9%	4%	7%	26%	25%	24%	22%	91%	89%
Poland	7%	6%	6%	5%	31%	28%	23%	20%	74%	73%
Spain	9%	11%	5%	8%	30%	28%	22%	21%	75%	74%
Sweden	11%	11%	9%	10%	15%	14%	14%	13%	92%	93%

Source: WHO, 2021.
