# Supplementary material for: Catastrophic health care expenditure among older people with non-communicable diseases in 11 European Union Member States
Source: PLoS One. 2026 Apr 29;21(4):e0346341. doi: 10.1371/journal.pone.0346341 (PMC13127918; doi:10.1371/journal.pone.0346341)
Supplement: S2 Table — (PDF) [file pone.0346341.s002.pdf]

S2 Table. Descriptive statistics for total sample (N=13.437)

	Frequencies	Mean	Minimum	Maximum
**Outcome variable**				
Catastrophic effects of health care expenditure for all sample with %10 threshold	1=catastrophic effect-yes (%13) 0=catastrophic effect-no (%87)	-	-	-
Catastrophic effects of health care expenditure for all sample with %25 threshold	1=yes (4%) 0=no (96%)	-	-	-
Catastrophic effects of health care expenditure for all sample with %40 threshold	1=yes= (2%) 0=no (98%)	-	-	-
**Explanatory variables**				
Number of chronic conditions	From 0 up to 5	1	0	5
Having one chronic condition	1=yes (52%) 0=no (48%)	-	-	-
Deceased by wave 8	1=yes (5%) 0=no (95%)	-	-	-
Ever smoked	1=yes (43%) 0=no (57%)	-	-	-
Alcohol consumption	From=0 not at all (0.3%) to 5=excessive consumption (%65)	-	-	-
Body mass index	From 27 up to 56	27	13	56
Physical activity	1=yes (81%) 0=no (19%)	-	-	-
High level of cholesterol	1=yes (25%) 0=no (75%)	0.25	0	1
Household size	From 0 up to 10	-	-	-
Gender	1=Male (%45); 0=Female (%55)	-	-	-
Age	From 66 up to 113	76	66	113
Children	From 0 to 13	-	-	-
Household Expenditure percentiles	From 1 to 5	-	-	-
Years of education	From 0 to 30	-	-	-
Country indicator Austria (N=583)	1=yes (% 4.3), 0=no (%95.7)	-	-	-
Country indicator Belgium (N=1600)	1=yes (% 12), 0=no (%88)	-	-	-
Country indicator Czech Republic (N=914)	1=yes (% 6.8), 0=no (%93.2)	-	-	-
Country indicator Denmark (N=1169)	1=yes (% 8.7), 0=no (%91.3)	-	-	-
Country indicator Italy (N=1625)	1=yes (% 12.1), 0=no (%87.9)			
Country indicator France (N=1088)	1=yes (% 8.1), 0=no (%91.9)	-	-	-
Country indicator Germany (N=920)	1=yes (% 6.9), 0=no (%93.1)	-	-	-
Country indicator Greece (N=1474)	1=yes (% 10.9), 0=no (%89.1)	-	-	-
Country indicator Poland (N=1230)	1=yes (% 9.1), 0=no (%90.9)	-	-	-
Country indicator Spain (N=1486)	1=yes (% 11), 0=no (%89)	-	-	-
Country indicator Sweden (N=1348)	1=yes (% 10), 0=no (%90)	-	-	-
**Non-communicable disease variable**				
Diagnosed diabetes or high blood sugar with the respondents	1=yes (17%) 0=no (83%)	-	-	-
Diagnosed cancer with the respondents	1=yes= (5%) 0=no (95%)	-	-	-

Diagnosed chronic lung disease with the respondents	1=yes (7%) 0=no (93%)	-	-	-
Diagnosed heart attack with the respondents	1=yes (16%) 0=no (84%)	-	-	-
Diagnosed stroke with the respondents	1=yes (5%) 0=no (95%)	-	-	-
Diagnosed high blood pressure with the respondents	1=yes (52%) 0=no (48%)	-	-	-

Note: Percentiles are based on total sample.
Due to the structure of the SHARE Wave 7 questionnaire, out-of-pocket expenditure data are only available for individuals aged 66 and above.
"Deceased by wave8" were generated by SHARE Wave 8 dataset.
