# Supplementary material for: Catastrophic health care expenditure among older people with non-communicable diseases in 11 European Union Member States
Source: PLoS One. 2026 Apr 29;21(4):e0346341. doi: 10.1371/journal.pone.0346341 (PMC13127918; doi:10.1371/journal.pone.0346341)
Supplement: S3 Table — (PDF) [file pone.0346341.s003.pdf]

S3 Table. Out-of-pocket payments for different types of services and different non-communicable diseases (all amounts are expressed in Euros)

Total Sample (N= 13.437)	Payments for inpatient care	Payments for outpatient care	Payments for prescribed drugs	Payments for nursing homes	Payments for aids, appliances and physical therapy	Dental payments annual (paid by yourself)
	Mean (SD)	Mean (SD)	Mean (SD)	Mean (SD)	Mean (SD)	Mean (SD)
Diagnosed diabetes or high blood sugar	14.35 (101.36)	159.08 (101.36)	215.05 (255.14)	222.32 (1217.46)	29.85 (134.64)	105.32 (532.31)
Diagnosed cancer	28.87 (123.14)	228.67 (506.41)	205.040 (234.95)	423.57 (4629.25)	29.46 (175.98)	145.59 (527.97)
Diagnosed chronic lung disease	24.60 (136.33)	214.62 (519.69)	252.57 (310.43)	312.37 (3768.20)	52.16 (231.94)	161.21 (655.234)
Diagnosed heart attack	32.65 (314.27)	186.76 (460.55)	235.09 (308.43)	403.02 (3823.66)	35.31 (162.68)	129.21 (594.16)
Diagnosed stroke	43.21 (265.32)	196.64 (439.84)	250.51 (292.47)	835.75 (6535.87)	46.25 (210.77)	142.08 (1110.00)
Diagnosed high blood pressure	13.81 (105.79)	199.35 (497.48)	185.29 (251.53)	169.09 (1652.579)	29.37 (159.16)	160.41 (978.66)
Having one chronic condition	18.91 (189.23)	192.79 (476.31)	186.15 (249.36)	201.40 (2001.163)	30.77 (163.23)	155.309 (948.093)
**Country Austria**						
Diagnosed diabetes or high blood sugar	29.60 (79.91)	233.83 (549.43)	224.77 (251.422)	620 (3535.59)	33.86 (111.68)	292.46 (1218.79)
Diagnosed cancer	150.39 (227.81)	213.62 (309.50)	302.41 (199.41)	96.87 (271.09)	61.87 (147.88)	42.37 (62.78)
Diagnosed chronic lung disease	79.03 (159.28)	366.86 (806.62)	295.94 (316.06)	331.67 (1262.49)	61.53 (201.91)	411.33 (1564.34)

	Payments for inpatient care	Payments for outpatient care	Payments for prescribed drugs	Payments for nursing homes	Payments for aids, appliances and physical therapy	Dental payments annual (paid by yourself)
**Country Austria (continue)**	Mean (SD)	Mean (SD)	Mean (SD)	Mean (SD)	Mean (SD)	Mean (SD)
Diagnosed heart attack	77.96 (184.01)	180.57 (460.07)	298.01 (283.29)	515 (2410.87)	47.07 (143.00)	181.63 (1095.29)
Diagnosed stroke	74.91 (115.96)	228.79 (560.05)	267.81 (263.43)	1065.15 (3421.77)	32.51 (95.22)	168.33 (545.74)
Diagnosed high blood pressure	44.04 (144.03)	260.14 (657.76)	204.03 (245.55)	141.88 (660.23)	47.43 (137.12)	225.55 (859.37)
Having one chronic condition	48.36 (145.88)	244.97 (627.26)	219.11 (254.50)	347.22 (2127)	45.70 (141.84)	215.55 (918.64)
**Country Germany**						
Diagnosed diabetes or high blood sugar	31.60 (73.66)	115.43 (253.45	146.65 (233.62)	70.82 (284.59)	43.273 (126.56)	86.58 (336.78)
Diagnosed cancer	79.09 (217.79)	108.94 (219.99)	171.17 (225.40)	437.12 (2949.59)	44.04 (143.87)	51.81 (122.82)
Diagnosed chronic lung disease	59.66 (200.97)	88.78 (190.12)	137.85 (170.47)	494.95 (2933.42)	46.03 (141.81)	44.86 (99.36)
Diagnosed heart attack	56.44 (126.94)	134.32 (344.09)	157.30 (243.97)	524.29 (3109.47)	51.14 (211.22)	109.29 (409.34)
Diagnosed stroke	86.61 (146.55)	68.14 (148.05)	139.01 (150.57)	788.44 (3754.68)	56.09 (164.26)	22.11 (85.10)
Diagnosed high blood pressure	39.37 (132.82)	133.75 (315.64)	112.67 (165.59)	140.69 (1231.02)	42.86 (154.58)	127.71 (529.23)
Diagnosed cancer	31.60 (73.66)	115.43 (253.45	146.65 (233.62)	70.82 (284.59)	43.273 (126.56)	86.58 (336.78)
Diagnosed chronic lung disease	79.09 (217.79)	108.94 (219.99)	171.17 (225.40)	437.12 (2949.59)	44.04 (143.87)	51.81 (122.82)
Having one chronic condition	41.92 (142.25)	126.16 (300.70)	118.04 (174.00)	181.95 (1508.01)	43.74 (153.75)	111.67 (473.93)

	Payments for inpatient care	Payments for outpatient care	Payments for prescribed drugs	Payments for nursing homes	Payments for aids, appliances and physical therapy	Dental payments annual (paid by yourself)
**Country Sweden**	Mean (SD)	Mean (SD)	Mean (SD)	Mean (SD)	Mean (SD)	Mean (SD)
Diagnosed diabetes or high blood sugar	23.15 (68.44)	260.30 (286.55)	150.62 (88.89)	217.23 (594.39)	11.90 (44.465)	223.45 (501.92)
Diagnosed cancer	15.74 (45.68)	250.76 (218.05)	142.89 (107.62)	190.56 (518.27)	6.66 (22.85)	156.25 (197.52)
Diagnosed chronic lung disease	22.33 (58.16)	361.28 (425.40)	171.25 (84.79)	218.18 (559.29)	18.56 (63.26)	338.22 (597.38)
Diagnosed heart attack	32.03 (81.94)	293.14 (325.77)	175.04 (107.01)	222.55 (577.14)	17.03 (64.86)	363.84 (1078.02)
Diagnosed stroke	55.09 (121.87)	313.87 (319.07)	157.33 (116.213)	282.53 (676.21)	14.60 (36.64)	181.92 (285.30)
Diagnosed high blood pressure	11.55 (45.09)	301.52 (343.33)	133.56 (99.39)	103.26 (412.13)	16.97 (73.82)	296.85 (637.514)
Having one chronic condition	15.76 (56.25)	302.04 (341.67)	138.80 (341.67)	138.80 (101.49)	129.10 (451.74)	15.63 (68.31)
**Country Spain**						
Diagnosed diabetes or high blood sugar	1.14 (18.56)	74.47 (308.83)	88.83 (122.78)	374.27 (1619.327)	26.43 (125.32)	108.14 (796.45)
Diagnosed cancer	0(0)	228.37 (817.88)	112.94 (149.07)	26 (115.23)	18.18 (120.60)	220.82 (818.94)
Diagnosed chronic lung disease	4.24 (35.72)	121.45 (392.64)	110.44 (138.04)	375.50 (1568.09)	67.21 (464.38)	96.48 (390.57)
Diagnosed heart attack	0 (0)	141.33 (511.89)	111.55 (138.83)	528.39 (1986.38	35.61 (149.76)	122.92 (509.52)

	Payments for inpatient care	Payments for outpatient care	Payments for prescribed drugs	Payments for nursing homes	Payments for aids, appliances and physical therapy	Dental payments annual (paid by yourself)
**Country Spain (continue)**	Mean (SD)	Mean (SD)	Mean (SD)	Mean (SD)	Mean (SD)	Mean (SD)
Diagnosed stroke	0 (0)	108.50 (282.50)	94.48 (119.18)	998.64 (2790.02)	81.35 (334.05)	675.13 (3942.617)
Diagnosed high blood pressure	0.54 (12.74)	152.93 (584.209)	89.38 (128.14)	273.80 (1258.58)	37.79 (233.52)	150.83 (761.17)
Having one chronic condition	0.41 (11.17)	141.99 (558.46)	89.27 (127.24)	312.47 (1450.34)	34.56 (210.58)	168.09(1129.84)
**Country Italy**						
Diagnosed diabetes or high blood sugar	2.17 (22.78)	401.37 (774.99)	226.77 (250.02)	335.38 (1575.53)	63.26 (249.69)	195.36 (672.61)
Diagnosed cancer	0 (0)	521.97 (844.56)	226.91 (295.86)	572.35 (2236.72)	76.79 (437.30)	236.78 (702.12)
Diagnosed chronic lung disease	0 (0)	460.35 (986.39)	278.58 (331.84)	70.56 (350.13)	84.97 (321.75)	229.46 (889.18)
Diagnosed heart attack	2.49 (24.37)	426.82 (912.146)	212.05 (276.23)	225.51 (1037.01)	32.43 (191.24)	231.66 (811.12)
Diagnosed stroke	0 (0)	455.64 (930.27)	209.15 (274.03)	417.91 (1485.07)	115.50 (438.88)	218.06 (835.16)
Diagnosed high blood pressure	0.98 (15.31)	459.57 (964.23)	190.32 (251.97)	124.92 (844.28)	38.85 (266.59)	396.65 (2196.88)
Having one chronic condition	0.80 (13.88)	426.91 (899.04)	185.38 (247.48)	165.80 (998.31)	41.48 (271.70)	346.41 (2000.32)
**Country France**						
Diagnosed diabetes or high blood sugar	11.28 (61.24)	117.03 (305.08)	56.89 (110.09)	265.55 (892.10)	32.74 (163.55)	63.84 (260.49)
Diagnosed cancer	12.80 (91.44)	332.42 (782.44)	45.07 (91.81)	2083.86 (13991.72)	22.42 (60.05)	286.69 (782.27)
Diagnosed chronic lung disease	5.20 (37.16)	132.66 (510.71)	93.76 (125.69)	2174.51 (13991.07)	26.43 (114.81)	98.43 (502.28)

	Payments for inpatient care	Payments for outpatient care	Payments for prescribed drugs	Payments for nursing homes	Payments for aids, appliances and physical therapy	Dental payments annual (paid by yourself)
**Country France (continue)**	Mean (SD)	Mean (SD)	Mean (SD)	Mean (SD)	Mean (SD)	Mean (SD)
Diagnosed heart attack	85.50 (802.95)	197.33 (593.83)	50.37 (85.98)	1762.98(11336.66)	23.63 (117.61)	142.55 (572.74)
Diagnosed stroke	43.85 (196.15)	170.235 (311.242)	52.86 (94.81)	6320.86(23166.18)	28.81 (85.66)	83.16 (233.32)
Diagnosed high blood pressure	26.66 (204.488)	136.68 (358.39)	67.95 (128.52)	663.47 (5855.871)	34.30 (158.92)	88.04 (312.23)
Having one chronic condition	45.06 (483.81)	170.80 (500.52)	66.08 (122.95)	687.18 (6455.071)	32.55 (155.613)	120.96 (476.02)
**Country Denmark**						
Diagnosed diabetes or high blood sugar	3.50 (34.30)	254.18 (369.20)	356.86 (351.05)	48.21 (262.53)	22.75 (79.13)	274.54 (585.84)
Diagnosed cancer	8.96 (60.12)	260.49 (361.43)	226.21 (212.39)	126.03 (645.93)	47.62 (167.37)	390.90 (935.45)
Diagnosed chronic lung disease	0 (0)	321.123 (578.06)	397.57 (389.82)	73.36 (359.53)	68.92 (230.10)	440.18 (1024.63)
Diagnosed heart attack	16.51 (176.27)	215.83 (330.28)	351.54 (345.92)	138.58 (489.15)	73.59 (247.71)	234.31 (529.86)
Diagnosed stroke	62.53 (309.56)	302.07 (495.81)	338.37 (346.72)	117.32 (466.42)	33.66 (102.86)	326.20 (630.24)
Diagnosed high blood pressure	1.85 (26.25)	260.88 (421.83)	254.02 (296.67)	72.81 (372.27)	43.19 (152.04)	324.95 (1192.39)
Having one chronic condition	6.43 (91.42)	250.80 (408.63)	260.05 (291.84)	72.95 (373.97)	45.94 (166.14)	319.81 (1121.80)
**Country Greece**						
Diagnosed diabetes or high blood sugar	17.29 (161.81)	153.20 (136.37)	239.26 (179.91)	143.83 (773.61)	14.39 (61.38)	22.91 (73.24)
Diagnosed cancer	0 (0)	203.52 (141.86)	284.92 (184.65)	112.10 (449.61)	4.28 (25.82)	13.67 (31.34)

	Payments for inpatient care	Payments for outpatient care	Payments for prescribed drugs	Payments for nursing homes	Payments for aids, appliances and physical therapy	Dental payments annual (paid by yourself)
**Country Greece (continue)**	Mean (SD)	Mean (SD)	Mean (SD)	Mean (SD)	Mean (SD)	Mean (SD)
Diagnosed chronic lung disease	54.35 (278.05)	139.78 (124.33)	237.31 (173.76)	93.88 (433.61)	25.11 (96.10)	14.55 (43.76)
Diagnosed heart attack	27.28 (187.39)	170.31 (144.64)	259.74 (183.57)	159.73 (742.70)	11.24 (56.75)	27.15 (76.77)
Diagnosed stroke	25.64 (226.45)	185.09 (143.79)	247.96 (159.49)	271.79 (1087.44)	20.24 (55.15)	11.28 (38.99)
Diagnosed high blood pressure	8.26 (112.76)	141.34 (126.79)	190.64 (151.16)	109.83 (653.25)	7.68 (41.98)	24.22 (59.91)
Having one chronic condition	9.28 (112.45)	143.53 (130.66)	192.3 (155.19)	97.64 (607.82)	8.30 (45.08)	24.51 (63.28)
**Country Belgium**						
Diagnosed diabetes or high blood sugar	51.01 (227.10)	153.40 (191.11)	388.86 (386.58)	364.95 (1209.35)	63.09 (162.01)	57.23 (467.76)
Diagnosed cancer	89.63 (214.78)	211.26 (336.42)	337.39 (324.74)	401.79 (1385.46)	41.11 (117.09)	69.20 (284.63)
Diagnosed chronic lung disease	55.09 (180.12)	207.70 (316.28)	418.45 (488.61)	260.79 (668.19)	99.80 (234.54)	79.85 (267.67)
Diagnosed heart attack	92.58 (505.38)	159.12 (179.69)	391.14 (339.26)	355.22 (1096.48)	107.54 (292.04)	70.24 (520.53)
Diagnosed stroke	140.86 (652.64)	200.16 (227.51)	462.61 (466.82)	574.03 (1659.56)	98.21 (254.01)	56.61 (147.41)
Diagnosed high blood pressure	44.48 (192.94)	182.59 (309.95)	322.14 (373.22)	311.71 (918.55)	62.25 (163.79)	92.82 (499.04)
Having one chronic condition	61.42 (345.69)	177.39 (284.76)	326.58 (371.60)	316.39 (943.22)	68.47 (206.46)	84.17 (481.53)

	Payments for inpatient care	Payments for outpatient care	Payments for prescribed drugs	Payments for nursing homes	Payments for aids, appliances and physical therapy	Dental payments annual (paid by yourself)
**Country Czech Republic**	Mean (SD)	Mean (SD)	Mean (SD)	Mean (SD)	Mean (SD)	Mean (SD)
Diagnosed diabetes or high blood sugar	8.94 (58.42)	35.16 (100.88)	106.69 (102.86)	81.29 (404.79)	7.94 (49.31)	35.76 (164.28)
Diagnosed cancer	24.02 (86.24)	22.16 (64.52)	112.46 (88.07)	109.95 (388.39)	0.33 (1.44)	20.91 (64.59)
Diagnosed chronic lung disease	11.01 (65.58)	24.72 (48.27)	87.52 (78.24)	4.71 (19.15)	3.58 (19.95)	17.53 (39.79)
Diagnosed heart attack	13.99 (65.80)	39.58 (117.75)	117.38 (108.91)	67.18 (312.65)	4.18 (31.34)	28.78 (103.44)
Diagnosed stroke	15.36 (62.66)	32.64 (116.99)	120.49 (109.88)	91.99 (452.47)	3.72 (17.00)	29.60 (116.92)
Diagnosed high blood pressure	7.47 (53.66)	41.28 (135.76)	89.93 (89.10)	50.91 (368.79)	5.43 (35.08)	44.04 (176.82)
Having one chronic condition	9.49 (59.38)	38.05 (127.30)	90.65 (91.53)	54.67 (368.90)	5.82 (39.19)	41.66 (179.87)
**Country Poland**						
Diagnosed diabetes or high blood sugar	0.49 (6.79)	32.73 (77.84)	396.76 (284.61)	12.57 (144.36)	10.55 (91.43)	12.73 (97.13)
Diagnosed cancer	0 (0)	30.77 (92.06)	356.03 (287.96)	2.00 (12.84)	0.29 (1.83)	20.63 (83.42)
Diagnosed chronic lung disease	0 (0)	29.43 (73.35)	355.66 (310.98)	3.56 (28.91)	25.87 (153.08)	5.01 (24.74)
Diagnosed heart attack	0.33 (4.82)	42.11 (96.22)	410.71 (539.10)	22.08 (186.01)	15.31 (94.96)	6.01 (28.13)
Diagnosed stroke	0 (0)	31.24 (59.73)	457.77 (323.59)	32.89 (254.74)	1.94 (7.35)	9.94 (42.07)
Diagnosed high blood pressure	2.86 (50.40)	44.58 (107.14)	350.84 (415.89)	11.38 (127.33)	11.32 (86.47)	18.37 (99.83)
Having one chronic condition	2.31 (44.31)	41.47 (104.28)	(335.59) (385.72)	12.39 (134.44)	9.83 (78.36)	16.62 (90.61)
